# Cutaneous Lymphoid Hyperplasia: A Case Report

**DOI:** 10.7759/cureus.90022

**Published:** 2025-08-13

**Authors:** Ibrahim Sulaiman, Faiza Al Raai

**Affiliations:** 1 Department of Pathology and Laboratory Medicine, Sultan Qaboos Hospital, Salalah, OMN; 2 Department of Dermatology, Sultan Qaboos Hospital, Salalah, OMN

**Keywords:** cutaneous lymphoid hyperplasia, forehead papules, immunohistochemical analysis, lymphoma, pulsed dye laser

## Abstract

Cutaneous lymphoid hyperplasia (CLH) is a localized reactive proliferation of polyclonal lymphoid cells in the skin. The condition can be idiopathic or result from various stimuli, with indolent nodular/papular lesions on exposed body areas. Despite its benign nature, it mimics cutaneous lymphomas both clinically and histologically. We present the case of a 56-year-old male patient who developed multiple erythematous papules and nodules on the forehead, gradually increased in size and number over a period of two months. Routine histological studies revealed dermal lymphocytic infiltrates, multiple lymphoid follicles with germinal centers containing tingible body macrophages. Immunohistochemical staining revealed a mixed population of B and T lymphocytes. The reactive germinal centers retained immunoreactivity for CD10 but did not show co-expression of BCL2. These findings confirmed the diagnosis of CHL. The patient was treated with three sessions of pulsed dye laser (PDL), followed by intralesional steroid and topical tacrolimus, with uneventful recovery after five years of follow-up. We also discuss the etiology, clinical features, diagnosis, and available management modalities for CLH.

## Introduction

Cutaneous lymphoid hyperplasia (CLH) is a reactive benign process histologically characterized by a dense collection of diverse polyclonal lymphocytes within the dermis [1], either idiopathic or secondary to different stimuli [1-6]. Clinically, it usually presents as a solitary or multiple dome-shaped eruption typically found on the head, neck, or trunk [2-4]. It may simulate cutaneous lymphoma (CL) in both clinical presentation and pathological findings [7,8].

One of the most challenging tasks in dermatopathology is distinguishing between benign and malignant lymphoid infiltrates. Cutaneous lymphomas (primary or secondary) also share many clinical and histopathological similarities with some inflammatory dermatoses. It is essential to distinguish between these illnesses accurately, as misdiagnosis can result in unpleasant health and psychological sequelae [2].

The pathologist must therefore be well-versed in dermatopathology and hematopathology, comfortably utilizing and exploring ancillary techniques, and, most importantly, willing to communicate effectively with the referring physician and thoroughly analyze clinical findings on a case-by-case basis when handling such cases.

## Case presentation

A 56-year-old man presented with a two-month history of forehead papules that had gradually progressed in size and number. He had no history of local trauma, insect bites, or pre-existing lesions at the site. He reported an adverse reaction to henna one year back, but no recent application of henna or hair dye. The patient had no other skin or systemic complaints. No personal or family history of connective tissue disorder. His medical history included type II diabetes, hyperlipidemia, and hypertension. He had been taking lisinopril, tenormin, metformin, and glimepiride regularly for several years with no reported side effects.

**Figure 1:**
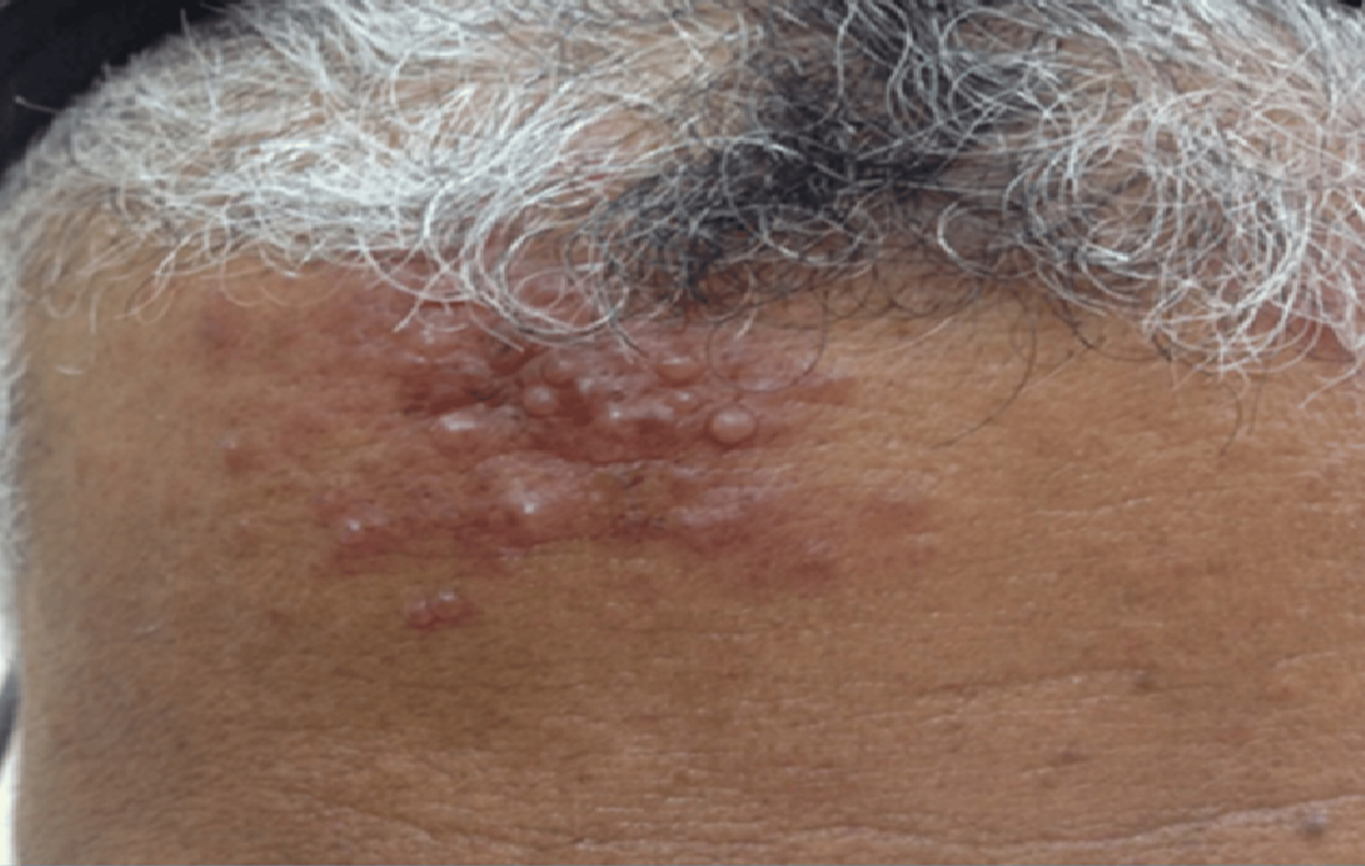
Multiple grouped erythematous papules and nodules on the forehead.

**Figure 2:**
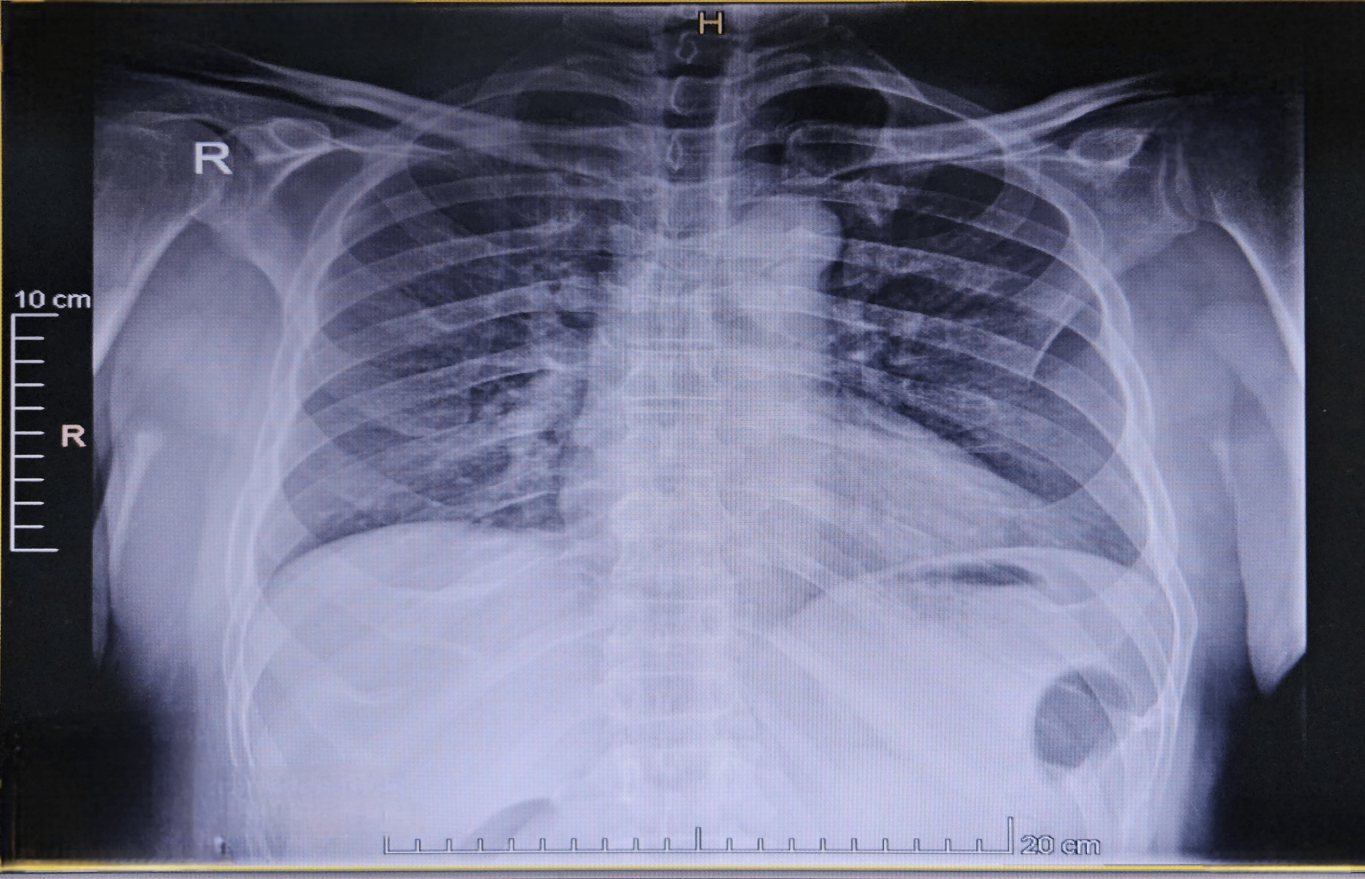
Chest X-ray with no significant finding.

Clinically, there were (5 cm) firm, non-tender grouped erythematous papules and nodules with no noticeable pigmentation, as shown in Fig. 1. No palpable local or general lymph nodes were observed. The results of the blood investigations (renal function test, liver function test, and C-reactive protein) and peripheral blood smears were unremarkable. The chest X-ray was normal (Fig. 2).

Initially, the lesions were treated for one month with a topical steroid (mometasone cream BID) and oral antibiotics (cephalexin cap. 500 mg TID for seven days). Nevertheless, follow-up revealed no improvement. Based on these findings, skin rosacea, lymphoma, pseudolymphoma, Jessner lymphocytic infiltration, and plaque form of polymorphous light eruption are among the clinical differential diagnoses. An incisional biopsy of the lesion was performed.

**Figure 3:**
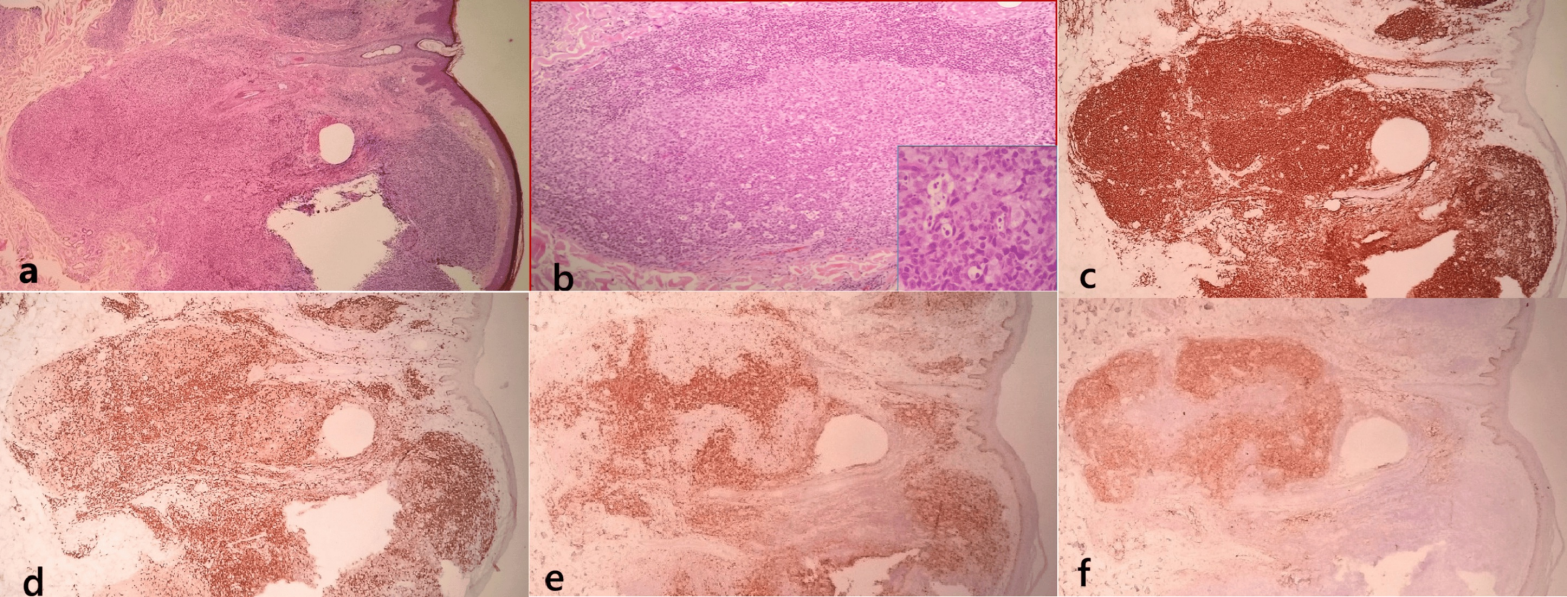
The pathological features of cutaneous lymphoid hyperplasia. (a) Dense, patchy, and nodular infiltration throughout the dermis (H and E, ×40). (b) Lymphoid follicle with well-formed germinal center (H and E, ×100). The inset shows a higher-power view of tingible body macrophages (H and E, ×400). Immunohistochemistry. (c) CD20+ B-cells (×40); (d) CD3+ T-cells (×40). (e) The germinal centers are negative for BCL-2(×40) (f) but positive for CD10 (×40).

Routine histological studies revealed dense, patchy, and nodular mid- and deep dermal infiltrates of small-to-medium-sized lymphocytes, plasmacytes, and histiocytes (Fig. 3a). Multiple lymphoid follicles with germinal centers containing tingible body macrophages were also observed (Fig. 3b). The infiltrate was separated from the epidermis by a Grenz zone.

The infiltrating lymphoid cells were medium-sized, with a high nuclear/cytoplasmic ratio and prominent nucleoli. Immunohistochemical staining revealed a mixed population of B (CD20) and T(CD3) lymphocytes (Fig. 3c, 3d, respectively). The reactive germinal centers retained immunoreactivity for CD10 (Fig. 3e) but did not show co-expression of BCL2 (Fig. 3f). They are also BCL6-positive, while negative for CD30 and MUM1 immunostains.

**Figure 4:**
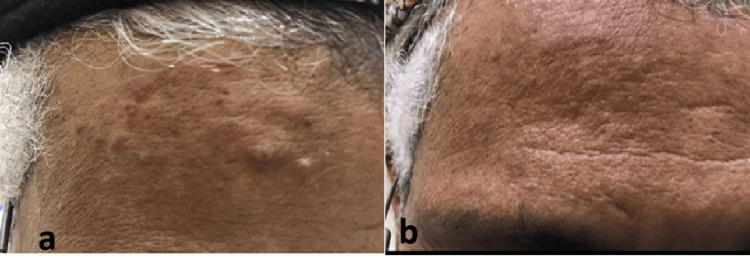
(a) The lesion showed a good response after the third session of pulsed dye laser (PDL) therapy. (b) Almost complete resolution after intralesional injection of triamcinolone and topical tacrolimus therapy.

These findings indicated benign CLH without evidence of a malignant neoplastic process. The patient was treated with three sessions of pulsed dye laser (PDL) therapy: the first session had a spot size of 7 mm, power fluence of 7 J/cm^2^, and pulse duration of 0.5 ms; the second session had a spot size of 7 mm, power fluence of 7 J/cm^2^, and pulse duration of 2 ms; and the third session had a spot size of 7 mm, power fluence of 7 J/cm^2^, and pulse duration of 2 ms (Fig. 4a).

No significant side effects were reported. Then, one session of intralesional corticosteroids, followed finally with topical tacrolimus 0.1% cream for three months, resulted in substantial improvement without recurrence after five years of follow-up (Fig. 4b).

## Discussion

CLH is a non-malignant reactive inflammatory phenomenon [7,9]. The terms "cutaneous pseudolymphoma," "lymphocytoma cutis," "lymphadenosis benigna cutis," "sarcoid of Spiegler-Fendt," and "cutaneous lymphoplasia" have also been used to describe this disorder [8,9].

The condition may be idiopathic or may arise as a secondary consequence of an array of inducing factors, including tattoo inks, immunizations, arthropod bites, scabies, metals utilized in body piercings, pharmacological agents, and infectious pathogens such as Borrelia burgdorferi, cutaneous leishmaniasis, herpes simplex, herpes zoster, and molluscum contagiosum viruses [5,6,10,11]. However, some elements that cause CLH are linked to cultural differences and vary by country [6].

With a slight female predominance, this illness primarily affects adults of a wide age range [6,9,10]. Although isolated, modest symptoms such as discomfort and pruritus may appear, the illness is primarily asymptomatic [3,9].

In terms of histology, CLH is identified by a noticeable nodular or diffuse infiltrate comprising a mixed population of small and large lymphocytes, accompanied by reactive follicles and tingible body macrophages within germinal centers, interspersed with histiocytes, plasma cells, and eosinophils. The infiltrate is typically localized within the dermis and hypodermis and is usually delineated by a grenz zone from the epidermis, devoid of atypical lymphocytes [2,6,9,10].

Immunohistochemical IHC analysis reveals mixed lymphocytes comprising CD20-positive B and numerous reactive CD3-positive T cells. The reactive germinal center cells express CD19, CD20, CD22, CD79a, Bcl-6, and CD10, yet they are negative for BCL2 and MUM1 immunostains. Germinal centers were sites of increased Ki-67 expression [2,6,8].

The differential diagnosis of CLH poses significant clinical and histopathological challenges. Therefore, it is imperative to differentiate it from CL [2,10,12]. This differentiation is typically accomplished through clinicopathological correlations, IHC investigations, and, in select instances, gene rearrangement analyses [6,9].

Marginal zone lymphoma is distinguished by the proliferation of small monocytoid lymphoid cells, the interspersed presence of plasma cells, and a lack of notable tingible body macrophages. Lymphoid cells were positive for CD20 and BCL2 and negative for CD10 and BCL6. Molecular analysis may reveal the translocation of t(11;18)(q21; q21) [13,14].

Follicular lymphoma occasionally involves the dermis with indolent progression. Histological analysis revealed a uniform population of centrocytes and centroblasts, with follicles lacking tingible body macrophages. The neoplastic cells were CD20, CD10, and BCL6 positive, with the co-expression of BCL2 within the neoplastic follicles (contrastingly absent in CHL). In complex cases where distinguishing between a reactive process and PCFCL (primary cutaneous follicular center lymphoma) is challenging, polymerase chain reaction may be employed to analyze immunoglobulin heavy-chain and J light-chain gene rearrangements [13,14]. 

The detection of atypical cells incites suspicion of mycosis fungoides. The atypical malignant T cells are positive for CD3 and CD4, exhibit a loss of pan-T-cell markers such as CD7 or CD5, and are also negative for CD20. Clonal rearrangement of T-cell receptor genes may be identified [13,14].

The first treatment recommendation is observation combined with a conservative approach because some cases have a benign course and resolve independently within weeks to months [4,10]. In instances arising from identifiable stimuli, the initial intervention involves the removal of the causative agent. Antibiotic therapy has demonstrated efficacy in cases attributable to infectious etiologies [9]. Occasionally, remission has been documented with incisional biopsy [3,12].

Many therapeutic modalities are available, including potent topical steroids, cryotherapy, thalidomide, interferons, localized radiation, dapsone, laser, antihistamine treatment, and surgical excision. Intralesional rituximab administration has been reported in patients with documented CD20+ lesions and a good response [6,9,10]. Anti-malarial drugs, methotrexate, triamcinolone acetonide, topical tacrolimus 0.1%, and cyclosporine have been reported to have variable success rates; however, these treatments may be considered in cases where total excision is impossible, residual, or multiple lesions [3,6,12].

In the present case, the lesions were initially managed with a topical steroid in conjunction with oral antibiotics due to the absence of any identifiable offending agents. This therapeutic approach yielded resolution in specific instances [1,2]; however, subsequent follow-up revealed no discernible improvement in our case. The incorporation of systemic oral steroid therapy, despite having been employed in select cases [7,12] with favorable outcomes, was not pursued in this instance owing to the patient's underlying diabetic background.

PDL technology has established a distinguished reputation for managing vascular lesions. However, according to a contemporary classification, its applications have been broadened to encompass nonvascular lesions, rendering it suitable for such interventions. It has been documented as a safe, well-tolerated, and efficacious treatment modality. PDL demonstrates effectiveness in addressing conditions without resulting in enduring scarring or hyperpigmentation. It can afford patients with both an aesthetically superior outcome and a reduced recovery duration [15-18]. Nonetheless, the substantial expense of the procedure may constrain its utilization, notwithstanding the outstanding aesthetic outcomes [15,19].

## Conclusions

CLH represents a distinct form of benign lymphoid proliferation. A final diagnosis requires clinical, histological, and immunophenotypic evidence. Pathologists are critical in synthesizing these diverse datasets to deliver precise diagnoses and inform therapeutic strategies. An incorrect diagnosis may precipitate inappropriate treatment modalities, underscoring the significance of a thorough diagnostic approach. The management of this benign pathological entity must be tailored to an individual patient.

## References

[1] Zhou LL, Mistry N (2018). Cutaneous lymphoid hyperplasia (pseudolymphoma). CMAJ.

[2] Charli-Joseph YV, Gatica-Torres M, Pincus LB (2016). Approach to cutaneous lymphoid infiltrates: when to consider lymphoma?. Indian J Dermatol.

[3] Mendoza Ramírez JB, Ayala D, Heald A, Moreno GY (2021). Differential diagnoses of pseudolymphomatous folliculitis: considerations as regards one case. BMJ Case Rep.

[4] Kakizaki A, Fujimura T, Numata I, Hashimoto A, Aiba S (2012). Pseudolymphomatous folliculitis on the nose. Case Rep Dermatol.

[5] Lee EH, Kim JY (2021). Cutaneous lymphoid hyperplasia developing on the site of a positive intradermal allergy test to cefotetan. Indian J Dermatol Venereol Leprol.

[6] Choi ME, Lee KH, Lim DJ (2020). Clinical and histopathological characteristics of cutaneous lymphoid hyperplasia: a comparative study according to causative factors. J Clin Med.

[7] Sepaskhah M, Yazdanpanah N, Sari Aslani F, Akbarzadeh Jahromi M (2020). Cutaneous pseudolymphoma as a rare adverse effect of medicinal leech therapy: a case report and review of the literature. Cureus.

[8] Khalil S, Donthi D, Gru AA (2022). Cutaneous reactive B-cell lymphoid proliferations. J Cutan Pathol.

[9] Lackey J, Xia Y, Cho S, Sperling L (2007). Cutaneous lymphoid hyperplasia: a case report and brief review of the literature. Cutis.

[10] Jain A, Majumdar B, Sen D, Sen S, Mishra P, Samanta A (2015). Asymptomatic papules over central and pericentral areas of the face. Indian Dermatol Online J.

[11] Recalcati S, Vezzoli P, Girgenti V, Venegoni L, Veraldi S, Berti E (2010). Cutaneous lymphoid hyperplasia associated with Leishmania panamensis infection. Acta Derm Venereol.

[12] Gutte RM (2013). Pseudolymphomatous folliculitis: a distinctive cutaneous lymphoid hyperplasia. Indian J Dermatol.

[13] Dewar R, Andea AA, Guitart J, Arber DA, Weiss LM (2015). Best practices in diagnostic immunohistochemistry: workup of cutaneous lymphoid lesions in the diagnosis of primary cutaneous lymphoma. Arch Pathol Lab Med.

[14] Skala SL, Hristov B, Hristov AC (2018). Primary cutaneous follicle center lymphoma. Arch Pathol Lab Med.

[15] Husain Z, Alster TS (2016). The role of lasers and intense pulsed light technology in dermatology. Clin Cosmet Investig Dermatol.

[16] Wang SP, Chang YJ, Chi CC (2017). Using pulsed dye laser to treat sebaceous hyperplasia: comparison of short and long pulse-duration pulsed dye laser. Dermatologica Sinica.

[17] Forbat E, Al-Niaimi F (2019). Nonvascular uses of pulsed dye laser in clinical dermatology. J Cosmet Dermatol.

[18] Nisticò S, Campolmi P, Moretti S (2016). Nonconventional use of flash-lamp pulsed-dye laser in dermatology. Biomed Res Int.

[19] Veitch D, Kravvas G, Al-Niaimi F (2017). Pulsed dye laser therapy in the treatment of warts: a review of the literature. Dermatol Surg.

